# Release of Monomers from Dental Composite Materials into Saliva and the Possibility of Reducing the Toxic Risk for the Patient

**DOI:** 10.3390/medicina59071204

**Published:** 2023-06-26

**Authors:** Soňa Tkáčiková, Ján Sabo

**Affiliations:** Department of Medical and Clinical Biophysics, Faculty of Medicine, Pavol Jozef Šafárik University in Košice, Trieda SNP 1, 040 11 Košice, Slovakia; jan.sabo@upjs.sk

**Keywords:** dental fillings, elution, HPLC analysis, saliva

## Abstract

*Background and Objectives*: The objective of this study was (1) to measure the amount of monomers released into the saliva depending on the time elapsed after the hardening of the composite and on the type of monomer used; and (2) with the prolongation of the light-curing procedure, to publish information on whether it would be possible to influence the level of leached monomers. *Materials and Methods*: HPLC technique was used to monitor the levels of the unpolymerized monomers Bis-GMA, Bis/EMA, TEGDMA, and UDMA from the four commonly used composite materials, released into the saliva of a volunteer with intact dentition. The levels were monitored in 3 time periods during 24 h after composite hardening. From every composite material, 4 samples were formed and cured with an LED lamp for 10 s, 20 s, 40 s, and 60 s. After the light curing, the same polishing procedure was used and the samples were leached in blank saliva samples. *Results*: We observed that every monitored composite material eluted monomers into the saliva after its application. The amount of monomers depended on the time elapsed after the curing of the composite and on the type of composite used. A 40 s LED curing procedure can reduce the amount of leached monomers in comparison with the standard 20 s procedure, especially for monomers of higher molecular weight. *Conclusions*: Our study confirmed the hypothesis that the release of monomers gradually decreases with increasing time after the hardening of the composite filling.

## 1. Introduction

Despite advances in dentistry, caries remains a serious global health problem. Poor oral health linked to systemic bacterial and inflammatory vulnerability can cause undesired outcomes, including uncontrolled diabetes, cardiovascular disease, and respiratory disease. It is known that heart attack and stroke are linked to bacterial infections and inflammation after other invasive treatments, likely because inflammation can damage the walls of arteries and contribute to the formation of plaques that clog arteries. The preservation treatment of teeth after the removal of damaged enamel and dentin continues with the reconstruction of the original anatomical shape of the tooth using filling materials. All dental materials must have suitable mechanical and physical properties, with emphasis on their biocompatibility with the human body.

Current composite materials are composed of a mixture of monomers, fillers, polymerization initiators, and other substances that improve their properties. The choice of monomers significantly affects the reactivity, viscosity, polymerization contraction, mechanical properties, and water absorption of the composite. In order to improve the application and mechanical properties of the composites, different monomers are combined into one composite. The composites are polymerized by visible light or chemically in situ. It is known that the degree of conversion (DC) is usually in the range of 55–77% [1,2,3].

The toxic effects of monomers have been confirmed by studies on several cell lines including human gingival fibroblasts. Alizadehgharib et al. reported that a combination of TEGDMA (triethylene glycol dimethacrylate) and UDMA (urethane dimethacrylate) had a synergistic proinflammatory effect on neutrophils by increasing the release of IL-8 and the formation of neutrophil extracellular traps, which may lead to altered inflammatory response and relate to previously reported adverse immune reactions caused by these substances [4]. The studies of Reichl et al. [5] and Manojlovic et al. [6] demonstrated that the sensitivity of different cell lines to different monomers is variable and correlates with monomer concentration and exposure time. Al-Hiyasat et al. [7] observed a 54.5% reduction in the pregnancy of mice after absorption of TEGDMA in the intestinal tract. Darmani et al. [8] pointed to a significant toxic effect of Bis-GMA (bisphenol-A-glycidyl methacrylate) and TEGDMA monomers on the reproductive organs of female mice after enteral application for 28 days. In vitro studies revealed that TEGDMA is considerably cytotoxic in various cell cultures, can easily penetrate membranes, and subsequently may react with intracellular molecules. Large deletions of DNA sequences caused by TEGDMA resulted in high mutation frequency. In addition, TEGDMA has been identified as an important resinous sensitizer in patients and professionals [9]. According to Gupta et al. [3] TEDGMA stimulates the growth of *S. mutans* and *S. salivarius* in a pH-dependent manner. In both in vitro and in vivo studies, it is confirmed that bisphenol A (BPA) acts as an endocrine disruptor, and therefore its direct use in dental materials is prohibited. Composites may contain BPA as an impurity from the synthesis process of Bis-GMA, but there are also indications that BPA may be released from composites following the degradation of Bis-GMA. Bis-GMA itself may induce the cytotoxicity and prostanoid production of pulp cells, leading to pulpal inflammation or necrosis via reactive oxygen species production. To monitor levels of the leached monomers Bis-GMA, TEGDMA, UDMA, and Bis-EMA (ethoxylated Bis-GMA), HPLC technique was used [3].

Our study aimed at determining by high-performance liquid chromatography (HPLC) the levels of unpolymerized monomers in the patient’s saliva in three time periods during 24 h after composite hardening and at finding out whether the prolongation of the UV curing procedure can influence the monomer leaching. Based on the obtained results, dentists should consider the possibility of reducing the intake of leached monomers by the patient. The null hypothesis was that a longer curing time would result in a smaller amount of leaching monomers. The second hypothesis was that there is an opportunity on the part of the patient to reduce the intake of monomers by spitting saliva shortly after the curing procedure. Adopting this practice would make sense only if the level of monomers will go down quickly after the hardening of the composite.

## 2. Materials and Methods

Saliva samples were taken from a volunteer with intact dentition by the passive drooling technique. The volunteer was instructed not to consume any food or liquids for at least 30 min before collection. The experiment was approved by the Ethics Commission UPJŠ LF (12N/2022).

The materials used for the experiment are commonly used for the dental fillings Tetric Prime Ivoclar, Mosaic, DentSply Sirona, and 3M Filtek. The characteristics of these materials according to their MSDS (Maintaining Safety Data Sheets) are presented in Table 1.

From each of the filling composites, 4 samples were prepared in the shape of a cylinder, with a height of approx. 2 mm and a diameter of 3 mm and weighing approximately 0.1 g. Subsequently, they were cured according to the procedure specified by the manufacturer, using a polymerization lamp Woodpecker LED B (Guilin, China) with a light power of 1200 mW.cm^−2^, 480 nm, and an irradiation time changing at 10, 20, 40, and 60 s from one side. Then the samples were brushed for 10 s with an Arkansas stone and for 10 s with an occlusal brush. All the materials were immersed in 1 mL of saliva and shaken in a thermomixer (Eppendorf, Hamburg, Germany) at 37 °C for 1.5 h. The composite materials were then removed from the saliva samples, and the samples were labeled 1-1 to 16-1 according to Table 1. The composite materials were then immersed in the fresh blank saliva samples and extracted for the next 4.5 h, so that 6 h elapsed from the filling formation (samples 1-2 to 16-2). Subsequently, the composites were removed from the saliva after 4.5 h and immersed in another portion of fresh saliva samples up to 24 h after formation (making samples 1-3 to 16-3). After the extraction time had elapsed, the composite materials were removed and the saliva samples were frozen at −80 °C immediately. The saliva samples were thawed before analysis. The proteins were removed by precipitation with acetonitrile (Merck, Darmstadt, Germany) and acidified with formic acid (Merck, Germany) to a final concentration of 0.1% in the sample. The samples were placed in a freezer at −20 °C for 60 min with regular vortex. The samples were then centrifuged for 20 min at 14,000× *g* (Boeco, Hamburg, Germany) at 4 °C. 500 µL of the collected supernatant was dried in a vacuum concentrator (Labconco, Kansas City, MO, USA) and dissolved in 100 µL of mobile phase A (MP A) before injection into the HPLC system. The samples were measured in duplicates.

### HPLC Conditions

Chromatographic analysis was performed using the HPLC system Agilent Technologies Infinity 1260 with a UV detector MWD VL (Agilent Technologies, Santa Clara, CA, USA) in gradient mode. Mobile phase A (MP A) was composed of acetonitrile–methanol (Merck, Germany)—water (Merck, Germany) in a volume ratio 1/1/3 *v*/*v*/*v*, and mobile phase B (MP B) acetonitrile–methanol in a volume ratio of 1/1 *v*/*v*. The flow rate was set to 0.6 mL.min^−1^. All the solvents were of gradient grade purity. The timetable for the gradient is presented in Table 2.

**Figure 1:**
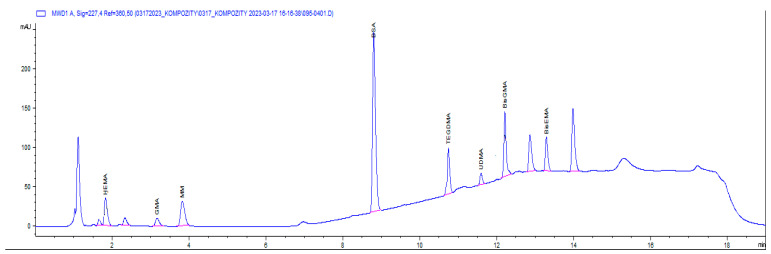
Chromatogram of the standard solution of dental monomers with a concentration of 10 µg·mL^−1^ in acetonitrile–methanol–water, 1/1/3 *v*/*v*/*v*, monitored at 270 nm.

Chromatographic analysis was performed at a controlled temperature of 40 °C on a Zorbax Eclipse AAA analytical column, 3 × 150 mm, particle size of 3.5 µm, Agilent Technologies. The sample injection volume was 20 µL. Stock solutions of monomer standards (Bis-GMA, Bis-EMA, UDMA from Sigma Aldrich, Darmstadt, Germany; TEGDMA from SPEX CertiPrep, Metuchen, NJ, USA) were prepared at a concentration of 1 mg mL^−1^ in methanol and stored in a freezer at −20 °C. They did not show degradation for at least 6 months. Working solutions were prepared from the stock solution by diluting into MP A in the range from 0.5 µg·mL^−1^ to 5.0 µg·mL^−1^. The analytes were detected by a UV detector at 3 wavelengths of 204 nm, 227 nm, and 275 nm; 227 nm was used for quantification. The calibration curve, constructed by the external standard method on the basis of the peak area, had a correlation coefficient in the range of 0.994–0.998. The extraction recovery was determined to be 95 to 100%. The detection limit (LOD) was determined as three times the detector baseline noise, and the quantification limit (LOQ) was calculated as ten times the baseline noise. The LOD and LOQ values of the individual monomer standards are given in Table 3, and the chromatogram of the standard solution with a concentration of monomers 10 µg·mL^−1^ in mobile phase A is presented in Figure 1.

## 3. Results

**Figure 2:**
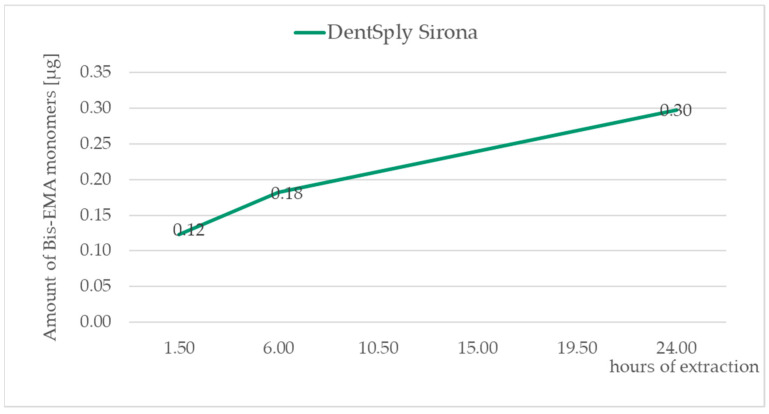
Bis-EMA monomer amounts released in µg from 0.1 g of the composite after 1.5, 6, and 24 h of extraction to the saliva sample after the 20 s curing procedure.

**Figure 3:**
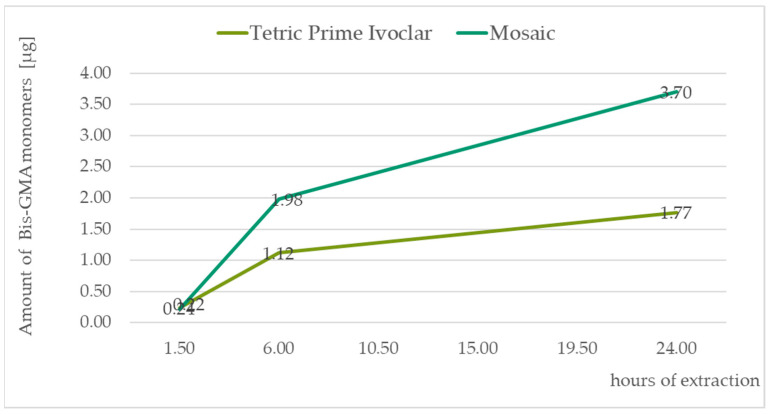
Bis-GMA monomer amounts released in µg from 0.1 g of the composite after 1.5, 6, and 24 h of extraction to the saliva sample.

**Figure 4:**
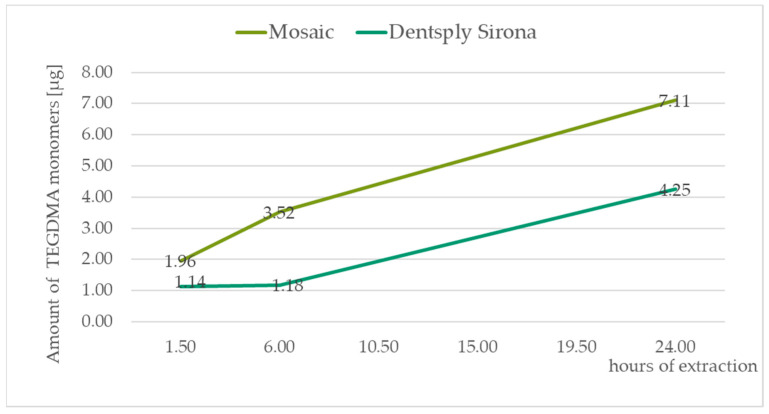
TEGDMA monomer amounts released in µg from 0.1 g of the composite after 1.5, 6, and 24 h of extraction to the saliva sample.

**Figure 5:**
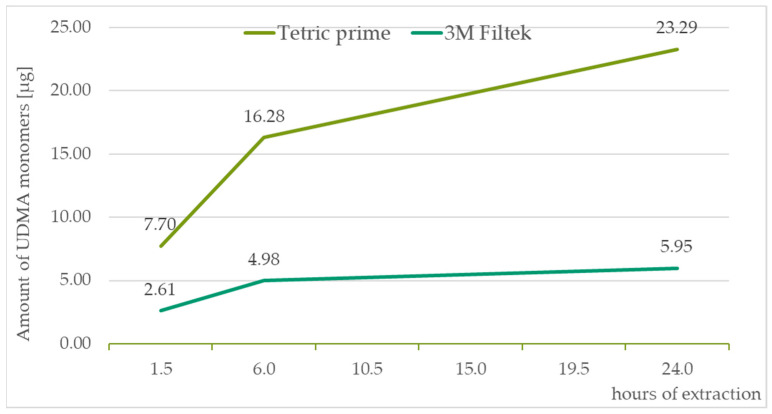
UDMA monomer amounts released in µg from 0.1 g of the composite after 1.5, 6, and 24 h of extraction to the saliva sample.

The identification of monomers determined by chromatographic analysis was according to the retention times of the standard solutions TEGDMA—10.7 min, UDMA—11.6 min, Bis-GMA—12.2 min, and Bis-EMA—13.3 min. All the measured values were recalculated to the amount of monomers released in μg from the composite material weighing 0.1 g. For graph presentation of monitoring the amount of monomers leached from a composite material as a function of saliva extraction time, samples with standard 20 s curation time were taken. The amount of eluted monomers in all three of the time segments are displayed in Table 4. Figure 2, Figure 3, Figure 4 and Figure 5 illustrate the amount of individual monomers released by composite fillings as a function of extraction time into saliva after a 20 s curing procedure.

## 4. Discussion

Bis-GMA-based dental polymer composites are a primary choice of dentists for anterior and posterior tooth filling due to their low polymerization shrinkage, volatility, and high viscosity. Materials containing UDMA have lower viscosity but higher toughness than Bis-GMA and can be used as their replacement. TEGDMA, a low-molecular-weight monomer, is used as a diluent in dental composites; reduces viscosity; and improves filler loadings, handling characteristics, and degree of conversion. On the other hand, TEGDMA increases polymerization shrinkage and water sorption. In comparison, Bis-EMA and Bis-GMA, the aliphatic chains in Bis-GMA, contain hydroxyl groups which the aliphatic chains in Bis-EMA do not have, and thus Bis-GMA is less flexible than Bis-EMA. The strong intermolecular bonds of Bis-GMA result in a decreased degree of conversion and crosslinking compared to Bis-EMA [10]. Our results showed that eluted levels of Bis-GMA are much higher in every time segment compared to Bis-EMA.

In summary, our experiment showed that the amount of unpolymerized monomers leached into saliva depends not only on the type of the monomer used but also on the producer. A significant difference was observed between the samples Dentsply Sirona and Mosaic in terms of the leached Bis-GMA monomer, where Mosaic eluted twice the Bis-GMA monomers every time. In both cases, we observed that the elution of Bis-GMA begins later after treatment, 6 h after application. After 24 h, the final amount counted was relatively low in comparison to the TEGDMA and UDMA monomers. In contrast, the TEGDMA and UDMA monomers were mainly eluted immediately after polymerization. The UDMA monomers eluted in higher amounts than the TEGDMA monomers in the first six hours. Our results showed that after a total of 24 h the level of the UDMA monomer in the eluate is much higher than in the other monomers. Comparing the molecular weight of the detected monomers, TEGDMA is the smallest one at 286,32 g.mol^−1^, followed by UDMA at 470.56 g.mol^−1^, Bis-GMA at 512.60 g.mol^−1^, and Bis-EMA at 568.70 g.mol^−1^. However, comparing the effect on the organism cytotoxicity and genotoxicity of this monomer, studies have shown that the toxicity increases from TEGDMA < UDMA < Bis-GMA˂ Bis-EMA [3,5,11]. These studies focused on their effects on basic cellular functions, inhibition of enzyme activities, disruption of cell morphology, membrane integrity, cell metabolism, and cell viability.

In the evaluation of the results, the influence of the curing procedure must also be taken into account. By comparing the different times of LED irradiation, we observed that the biggest differences are between 10 s and 20 s at every monomer. Prolongation of curing time from 40 s to 60 s lowers the amounts of monomers. Ak et al. tested the curing of resin-based materials at 20 s and 40 s for the LED unit and at 40 s curing time for the halogen light for the 1 mm thickness. For the sealant groups, polymerization with LED for 40 s yielded less monomer elution than the recommended 20 s. For the composite resin groups, the recommended 20 s curing time for Filtek Z250 resulted in the same amount of elution compared to 40 s of curing with the LED and the halogen light. However, 40 s of curing with the LED eluted less in comparison to the halogen at 40 s [12]. This conforms to our measurements with the LED lamp, where we observed that a longer time of curing resulted in lower amounts of leaching monomers. The difference between 40 s and 60 s did not present as significant a difference in leaching amount as between 20 s and 40 s. Lempel et al. analysed the correlation between the quantity of eluted monomers from a dental resin-based composite using reverse-phase HPLC and the DC using micro-Raman spectroscopy. They observed that there was a direct proportion in the DC and an inverse proportion in the monomer elution when the energy of light polymerization was increased from 20 to 40 J cm^−2^; however, a further increase in energy density did not influence the DC significantly. They also tested a 1 mm composite layer increment up to 3 mm, and it led to a 10% decrease in DC and a 30–35% increase in monomer elution. A further increase in depth from 3 to 4 mm caused a 30% drop in DC and a 55% increase in the amount of leached monomers. The limitation of our experiment was that the washing process of monomers from the composite material took place over its entire surface. In a real situation, part of the dental filling is not in contact with saliva but with dentin. However, some studies highlight the possibility of the passage of monomers into the patient’s body through dentin [13,14]. A meta-analysis by Van Landuyt et al. [15] based on data from 72 scientific articles confirmed a statistically significant correlation between the volume of the extraction solution and the amount of released monomers. Another limitation of our experiment was that it was not performed with a continuous supply of fresh saliva during elution but only in three selected time periods. The real situation in the oral cavity represents a system with a continuous supply of solvent–saliva, so the amount of released monomers can be higher.

It is clearly shown that a certain quantity of monomers is always eluted from composite fillings into saliva. Unlike toxic reactions, allergic reactions are not dose-dependent. The latter could occur with low-concentration exposures to substances released from dental materials. In a study on reported side effects, it was found that around 16% of the patients reported that they reacted to a composite, while over 50% of the dental personnel reacted to the composite [16]. There are two kinds of lymphocytes: B-cells and T-cells. B-cells produce antibodies, which are proteins that bind to and destroy or neutralize antigens. T-cells do not produce antibodies; instead, they bind directly to an antigen and stimulate an attack on it. Allergic reactions can have immediate or delayed effects depending on whether the antigen triggers a response by B- or T-cells [17]. Oral lichen planus is a chronic inflammatory condition in the oral mucosa caused by an immunological mechanism [18]. Inflammation is the trigger of the early phases of the atherosclerotic process, and an increase in inflammatory cytokines is associated with a higher risk of developing cardiovascular diseases [19]. So it is crucial to eliminate amounts of leaching monomers as much as possible. Sasaki et al. [20] found that gargling with warm water at 37 °C for 30 s after filling can reduce the amount of leached BPA by more than 10 times, depending of course on the composition of the filling material. Rueggeberg [21] and Komurcuoglu [22] demonstrated that when the surface of the filling was hand-scrubbed with pumice with either a prophy cup or a cotton roll this resulted in the lowest amount of residual monomeric content. However, they admitted that none of the surface treatments eliminated the surface residual monomeric content. With our experiment, we showed that prolongation of the curing procedure from 20 to 40 s can lower the amount of monomers. Based on our measurements, TEGDMA and UDMA monomers are washed out the most in the first hour after application. Thus, saliva spits outperformed in the first minutes after the application of composite should partially reduce the amount taken in by the saliva. Using warm water for the mouth rinsing can enhance its effectiveness.

On the other hand, it is well known that the aging of composite materials also leads to the increased release of unpolymerized monomers that were initially trapped in the polymer network [23,24]. In our future research, we want to perform an overview of the residual amounts of monomers in the saliva of dental patients with the help of the LC-MS/MS method. Based on the medical documentation with the cooperation of the dentist, we would look for the age of the patients’ fillings and detect if there is a relationship between this and the potentially detected released monomers. If a correlation occurs, monitoring the presence of monomers in the patient’s saliva during a preventive dental examination can be used as a diagnostic tool for the wear of dental fillings and thus indicate which filling needs to be replaced with a new one.

## 5. Conclusions

The availability of independently obtained data on the amount of monomers released from a commercially produced composite into saliva during the first 24 h after application may help clinicians and patients in the further selection of a suitable dental restorative material. Moreover, it can be useful for the optimization of polymerization conditions, as they are influenced by several factors such as the wavelength and intensity of the LED source, the duration of exposure, the restoration thickness, and the light source distance. Gűzel et al. [25] confirmed that different curing methods influence the amount of released monomers. Our results indicated that not only the curing procedure but the composition of the filling itself can significantly affect the amount of eluted monomers.

## Figures and Tables

**Table 1:** Overview of composite materials used to create the analyzed samples.

Sample Number	Product Identifier	LOT Number	Manufacturer	Monitored Monomers
1, 2, 3, 4	Tetric Prime Ivoclar	Z00RW7	Ivoclar Vivadent AG, Schaan, Liechtenstein	UDMA Bis-GMA
5, 6, 7, 8	Mosaic	BKMWT	Ultradent Products, Inc. South Jordan, UT, USA	TEGDMA
				Bis-GMA
9, 10, 11,12	DentSply Sirona	2109000860	DentSply Bensheim, Germany DeTrey GmbH Konstanz, Germany	TEGDMA Bis-EMA
13,14,15,16	3M Filtek	NA52898	3M,	UDMA
			Maplewood, MN, USA	

**Table 2:** Gradient elution timetable used for chromatographic separation of monomers Bis-GMA, Bis-EMA, TEGDMA, and UDMA by HPLC/UV.

time [min]	0	1.5	10	15	15.5	20
% MP B	0	0	80	80	0	0

**Table 3:** Detection limit (LOD) and quantification limit (LOQ) values for the determination of monomers in saliva by HPLC/UV at 227 nm.

	Bis-GMA	Bis-EMA	TEGDMA	UDMA
LOD [µg·mL^−1^]	0.003	0.037	0.005	0.0134
LOQ [µg·mL^−1^]	0.0104	0.123	0.0166	0.044

**Table 4:** Amount of monomers eluted from samples 1–4—Tetric Prime Ivoclar, 5–8—Mosaic, 9–12—DentSply Sirona, 13–16—3M Filtek. Samples 1-1 to 16-1 represent amount of monomers leached after 1.5 h extraction; samples 1-2 to 16-2 represent amount of monomers eluted after 6 h of composite formation; and samples 1-3 to 16-3 amount of monomers eluted after 24 h to fresh saliva. The given amounts of monomers are based on a filler weight of 0.1 g. Standard deviation (SD) is obtained from 2 measurements.

Sample	TEGDMA [µg]	SD [µg]	UDMA [µg]	SD [µg]	Bis-GMA [µg]	SD [µg]	Bis-EMA [µg]	SD [µg]
1-1			9.087	0.282	0.244	0.006		
2-1			7.704	0.332	0.241	0.005		
3-1			8.991	0.371	0.243	0.005		
4-1			9.111	0.409	0.242	0.011		
5-1	1.684	0.004			0.195	0.007		
6-1	1.957	0.183			0.216	0.008		
7-1	1.200	0.024			0.148	0.012		
8-1	0.961	0.029			0.150	0.013		
9-1	1.297	0.026					0.109	0.005
10-1	1.136	0.16					0.123	0.014
11-1	1.497	0.058					0.063	0.002
12-1	0.845	0.025					0.065	0.003
13-1			2.965	0.118				
14-1			2.606	0.071				
15-1			2.229	0.124				
16-1			1.859	0.065				
1-2			9.067	0.618	0.885	0.008		
2-2			8.580	0.118	0.881	0.034		
3-2			8.514	0.011	0.884	0.017		
4-2			6.205	0.077	0.669	0.014		
5-2	1.916	0.012			1.777	0.003		
6-2	1.566	0.030			1.762	0.128		
7-2	2.610	0.064			1.706	0.029		
8-2	1.588	0.073			1.321	0.004		
9-2	0.061	0.001					0.068	0.020
10-2	0.044	0.003					0.059	0.022
11-2	0.024	0.003					0.058	0.017
12-2	0.022	0.007					0.051	0.015
13-2			2.439	0.041				
14-2			2.372	0.004				
15-2			1.881	0.078				
16-2			1.562	0.047				
1-3			7.120	0.697	0.661	0.012		
2-3			7.001	0.610	0.646	0.014		
3-3			6.087	0.544	0.624	0.077		
4-3			4.810	0.417	0.631	0.001		
5-3	3.312	0.342			1.533	0.115		
6-3	3.582	0.352			1.723	0.037		
7-3	4.022	0.303			1.210	0.132		
8-3	2.822	0.096			1.190	0.111		
9-3	2.048	0.301					0.144	0.024
10-3	3.070	0.418					0.116	0.010
11-3	1.219	0.058					0.075	0.000
12-3	1.159	0.034					0.045	0.002
13-3			1.234	0.002				
14-3			0.976	0.030				
15-3			0.870	0.060				
16-3			0.746	0.006				

## Data Availability

Not applicable.
